# New York Pathological Society

**Published:** 1881-01

**Authors:** 


### Reports of Societies.

### NEW YORK PATHOLOGICAL SOCIETY.

*Strangulated Hernia.*—Dr. J. W. Howe presented a specimen of strangulated hernia with the following history: L. M——, a tailor, aged fifty-one years, was admitted to St. Francis' Hospital, October 15th, suffering from a strangulated oblique inguinal hernia. Twelve months previous to admission, while lifting a heavy tailor's iron, a tumor made its appearance in the left inguinal region and scrotum. The contents of the tumor was replaced by the patient without difficulty, and there was no further symptoms of hernia until the day before his admission to the hospital, when, from the same cause as before, the tumor reappeared. This time the tumor was painful, and could not be reduced by the patient.

A physician was summoned who applied warm water and chamomile leaves, followed by taxis, but without avail. At the end of twenty-four hours the physician applied a truss and sent the patient to the hospital, unrelieved of strangulation.

On admission the patient complained of great pain over the tumor, particularly when the latter was pressed upon. He had had no passage from his bowels since the previous morning, nor had his bladder been emptied during that period. The abdominal cavity was distended, and gave a dull percussion sound below, and contained fluid. The pulse was small, irregular and frequent. All the symptoms were much more grave than were usually witnessed in a strangulated hernia of twenty-four hours' duration. He had no stercoraceous vomiting; indeed, the patient seemed too weak to vomit. After etherization the sac was opened in the usual manner. A large mass of coagulated blood was found covering the intestines, the results of the taxis and ignorant application of the truss. The structure was tight, and when cut a large quantity of serum—over a gallon—escaped from the abdominal cavity. The intestine was much bruised at one point, but in other respects was not injured. It was returned to the abdominal cavity, and the usual dressing applied. Death took place twenty-four hours after the application.

The autopsy was made by Dr. Wendt, curator to the hospital. The cavity of the abdomen contained a large amount of brownish colored fluid. The intestines were congested, and were loosely matted together by pseudomembrane. A piece of intestine, six inches long, and more congested than the rest, was covered with purulent false membrane. An oblique line of ecchymosis was found in the serous coat of this part extending about two inches into the mesentery, also large and small ecchymoses on its mesenteric attachment. The visceral and parietal peritonitis about the cut or the internal ring. Both kidneys were congested and the spleen was in the condition of perisplenitis. The liver was in the atrophic stage of cirrhosis.

The specimen was presented not so much on account of its pathological as its clinical interest, as it illustrated a method of treatment which was not uncommon among ignorant practitioners.

Dr. Carpenter thought that the clinical features of unusual interest in the case were the sudden occurrence of a hernia, which filled the scrotum, and its easy reduction by the prtient.

*The bad effects of unskilful taxis.*—Dr. Briddon remarked that its sudden occurrence might be explained by a patent condition of the tabular vaginal process of the peritoneum, except at the internal ring, the partition at the latter point giving way and allowing the hernia to descend directly into the scrotum. But such herniæ were attended with grave symptoms from the first, and were difficult of reduction. He thought death in Dr. Howe's case was due to delay in the operation and to the injuries inflicted during taxis. As a rule, he thought taxis did harm in such cases, and that it was much bettter to perform the operation without delay.

Dr. Gibney asked if taxis was not usually attended with good results.

Dr. Briddon replied that he believed taxis, as a rule, did more harm than good in cases of strangulated hernia.

Dr. Howe believed in taxis before the operation, especially by the gravity method of Hamilton.

Dr. Ripley did not think that taxis should be abandoned because it had been abused. Every physician was in the habit of reducing strangulated herniæ by taxis, and the patients recovered. Sometimes ether was administered to that end, but not always.

Dr. Briddon said that he did not condemn the method, but the ignorant men who practiced it.

Dr. Heinemann, in regard to the time which might elapse between the occurrence of a strangulated hernia and death therefrom, stated that he had performed an autopsy at the end of strangulation. The patient was brought into the hospital at one o'clock of the day of his death, and died three hours afterwards, and after an operation had been attempted. A portion of the ileum was constricted, and one end of the gut was floating free in abdominal cavity, and had discharged its contents in said cavity.

Dr. Peabody referred to the case of a gentleman with strangulated hernia who died after taxis had been employed. At the autopsy it was found that the peritoneum above the internal abdominal ring had been separated from the transversalis facia, and more than a foot of the ileum had been forced into the space thus created. There was a tight constriction found at the neck of the sac.

*Exsection of ankle.*—Dr. Howe presented a second specimen, consisting of two inches of carious tibia and fibula, with a small portion of the carious astragalus, which he removed by exsection from a man, aged forty-four years, a patient in Charity Hospital. The disease was of over a year's duration, and, at the end of the operation, the patient was much reduced by pain and suppurative discharge through openings in the neighborhood of the ankle. Notwithstanding his weakened condition, the operation was performed with good result, the patient having a useful limb.

*A Fatty Ovarian Cyst.*—Dr. Garrigues presented a specimen of fatty deposit in an ovarian cyst. It was sent to him by Dr. Dawson, who had removed it after the patient's desth. Only a portion of the cyst-wall could be obtained, but sufficient to show the condition named. The cyst itself was of the mixed variety. The fatty deposit was exclusively in the outer layer of the cyst, and was for the most part lobulated in character. At one point there was a diverticulum from the cyst-wall the size and shape of the finger of a glove. It presented an appearance worthy of note, and was of special interest in regard to the question of its origin. Dr. Garrigues stated that he had not been able to find another case on record of adipose deposit in an ovarian cyst. Dr. Noeggerath had informed him that he (Dr. N.) had met with such a condition in ovaries removed by Battey's operation. He further remarked that ovaries covered with adipose tissue were normally found in the cat.

Dr. Briddon thought that the finger-like process was a secondary cyst which had become contracted.

Dr. Peabody remarked that the adipose tissue over the cyst-wall looked like that of portions of the omentum that might have become adherent.

Dr. Garrigues said that the tissue was positively not omentum, as there were no adhesions of the latter to the tumor found post mortem. The elongated cyst was probably altered by contraction, but its shape was quite remarkable.

*Diphtheria and Acute Emphysema of Lungs.*—Dr. Tauszky presented the larynx and trachea of a child upon whom tracheotomy had been performed for membranous croup· The patient did well for twenty-four hours, when it was seen by Dr. Ripley, who gave an un'avorable prognosis. The condition of the lung found corroborated the statement of Dr. Ripley, in regard to these cases, that they do not die of pneumonia, but of an extension of the membrane downward producing acute emphysema, or hyperinflation of the lung rather than consolidation.

Dr. Tauszky b·lieved that the case was one of membranous croup rather than of diphtheria, as the submucous layer was not involved in the deposit. The specimen was, on motion, referred to the Microscopical Committee.

Dr. Peabody exhibited sections of the brain, liver, and kidney removed from the body of a patient who died in an attack of congestive pernicious remittent fever. The points of interest were the deep pigmentations to be seen along the lines of the vessels of the specimens and in the vessels themselves. Some of the latter vessels, especially in the brain, were completely filled with pigmented material.

Dr. Briddon presented a large interstitial fibroid of the uterus removed by abdominal section. This was accompanied with a written history.

*Tape-worm.*—Dr. Van Giesen exhibited a specimen of tape-worm which had been discharged from a German midwife who had used, according to his directions, the fluid extract of aspidium marginale. Not giving the doctor an opportunity of examining the fæces the head of the worm, which should have been in them, was not found. A teaspoonful of the following mixture was taken six times at intivals of every fifteen minutes: Fluid extract of aspidium, ʒ ij., olive oil, ʒ iv., sulph. ether, ʒ ss. This was followed by an active purge of castor oil and croton oil. The following morning the worm, measuring twenty-eight or thirty feet, was discharged.

*Diphtheria.*—Dr. Van Giesen also presented a specimen of descending laryngeal diphtheria, which had been removed from a female child aged twenty-one months. He first saw the case October 20th. At that time the patient had a slight amount of what was conceded to be diphtheritic membrane in the lower part of the pharynx. She was otherwise comparatively well. There was no increase of temperature, and no great increase in the frequency of the pulse. The child apparently got better, and when there was no visible membrane in the pharynx, the case was dismissed simply with the precaution added, that the child might be taken worse at any time. On October 26th the patient became very hoarse, and was unable to make anything more than a whispering cry. The respiration was normal, the throat was very little reddened, and by a very prolonged examination nothing could be seen in the pharynx. The remedies which had already been prescribed were repeated. The breathing became much embarrassed on the 27th, and small doses of turpeth mineral were administered. The vomiting that followed the second dose brought up a membranous cast of the trachea (which was exhibited). The breathing was very much relieved, and inhalations of solution of bromine with small doses of the latter were given. The child died twenty hours after with symptoms of suffocation.

Dr. Van Giehen remarked, in conclusion, that he had been unable clinically, pathologically, or microscopically to distinguish between diphtheria and membranous croup.

*Retro-Peritoneal Sarcoma.*—Dr. Heinemann exhibited a specimen of retro-peritoneal sarcoma, with the following history:

Mary Fischer, aged twenty-two years, New York, single. Family history good. Well up to one year ago, when exposure, just before a menstrual period, caused a permanent cessation of the menses. She subsequently complained of slight discomfort about the left lumbar region; continued in a rather uncertain state of health up to four months before death, when dyspnœa set in; she begun to fail and suffer from disturbed digestion. She gradually became weaker, and dyspnœa became more severe, her abdomen enlarged, and rapidly within the last fortnight; ascites and slight jaundice followed. The urine was constantly loaded with urates. The patient was seen October 28th and November 5th by Dr. G. G. Wheelock, and was attended by Dr. Bleything. On account of the extensive dulness over the region of the spleen and the anæmia, it was thought a possible case of leucocythæmia, and when the subsequent rapid enlargement of the liver occurred, malignant disease of the liver was suspected. The patient died of asthenia, November 12th, 1880

*Autopsy*, made by Drs. Wheelock, Bleything, and myself.—Body moderately emaciated, jaundiced, marked prominence of epigastric region.

Heart and lungs normal. Liver, weight ten and three-fourths pounds, extended above from the right third intercostal space to the lower border on the third rib of the left side, downward to the upper portion of the hypogastric region. The right lobe was enormously increased in size and invaded by numerous large and small new-growths. The left lobe was normal in size and contained a few small new-growths.

Stomach was small, and displaced towards the left; the transverse colon was pushed down to the hypogastric region. Spleen and kidneys were normal.

In the left lumbar region, and apparently intimately connected with the kidney, but subsequently found to be independent, is a large tumor weighing not quite four pounds, which, upon section, seemed to be composed of small and large alveoli, filled with yellowish, firm, and in some places red marrow-like material, and infiltrated in parts with calcific deposit.

Mesenteric and lumbar glands were much enlarged and infiltrated.

Microscopic examination reveals the tumor to be a large-celled retro-peritoneal sarcoma.

*Dysentery; Suppurative Hepatitis: Perforation Through*

*into the Right Pleural Cavity ; Pericarditis and Pleurisy with Effusion.*—Dr. Satterthwaite presented the following case as an instance of hepatic disease dependent on dysentery, a condition which he was disposed to think was much more common in this latitude than was usually supposed:

H. V. ——, fourteen, office boy, was admitted into St. Luke's Hospital October 25, 1880 The history he gave was as follows: In July last he had an attack of 'cholera morbus," which lasted about ten days. After feeling better he returned to his work, and for a week was able to attend to his regular duties. Then he was attacked with what was thought to be "dumb ague." He had chilly feelings, felt sleepy, and wanted to sit by the fire. After a while his breathing became difficult, and he had cough In September last, about the middle, he began to have night-sweats, and he lost flesh. There was pain in the right side, of a dull character, shooting up to the corresponding shoulder. When admitted into the hospital the abdominal veins had become distinct, and the liver was found to be enlarged and tender. There was also some tympanitis, but no vomiting. Night-sweats continued. On November 8th the patient was taken suddenly with extreme dyspnœa. Dulness was found over the entire left side of the chest, and the left lung was œdematous. The patient died at 5:30 P.M. of the same day.

At the post mortem examination the liver was found, by measurement, to be enlarged, and the seat of many abscesses, one of which had burst through the diaphragm and discharged into the right pleural cavity. Other abscesses, through their contiguity with the peritoneal cavity, had set up a peritonitis of subacute character.

In searching for the cause of the suppuration there was little difficulty in associating it with a concomitant lesion, viz., dysentery. The mucous membrane in the rectum, sigmoid flexure, and descending colon was marked by small ulcers that had nearly healed, and were evidently dysenteric in their nature.

The empyema, excited and augmented by the discharge of pus and necrotic tissue from the liver, had caused the right lung to be so compressed against the spinal column that the respiratory function upon that side was abolished. The sudden œdema of the opposite lung was thought to be similar in character to a like condition often observed in pneumonia, where, after an inflammatory process had been proceeding slowly within one pleural cavity, it suddenly shifts to the other, causing congestion, œdema, and often a rapidly fatal issue. An additional cause of sudden death in this case may have been the effusion within the pericardium and left pleural cavity, which added further to the difficulty of breathing.

Dr. Satterthwaite stated that recently he had seen several cases of hepatic suppuration, and had been induced to consult somewhat hurriedly the records of a few New York hospitals. In this way he had collected six cases from the St. Luke, Presbyterian, and Mount Sinai hospitals, and these, together with twenty-six from the Transactions of the Pathological Society, made a total of thirty-three fatal cases. From them it appeared that the most common cause that had been ascertained was dysentery; the next most frequent was associated with the irritation produced by the presence of calculi in the gall-bladder or biliary passages; medullary cancer, traumatism and operation in or about the pelvic fossa held more subordinate places. These statistics differed from the recent ones of Baerensprung, given by Thierfelder in the last number of "Ziemssen's Cyclopædia, where, in a total of 108, sixty-eight were associated with traumatisms, of which thirty were pyæmic. In other respects the statistics agreed in so far that affections along the line of the portal system held the next place in the rank of causes, and ulceration of bileducts the third position in order. Pyæmia was observed much less frequently in hospital practice than formerly, and hepatic abscesses of pyæmic origin were quite rare.

In the majority of cases (about two-thirds), the abscesses were single, and these were located, usually, in the right lobe. The most frequent assigned cause of death was peritonitis, due to an extension of the disease either from the liver or biliary system. Some urinary complication, to which the convenient name of Bright's disease was given, came in for an occasional cause of death—certainly albumen was by no means rare, and casts were occasionally found, while the specific gravity was apt to be low and suppression not infrequent at the final issue. In the present instance the rupture into the pleural cavity was a rare incident in the disease, though rupture in the lung was not so uncommon. While not frequently symptoms were found in a certain number of cases, and they are given in the order of relative frequency: 1, rigors and sweats, often under the name of "chills and fever;" 2, pain in epigastrium; 3, jaundice; clay or, occasionally, bronze-colored stools. Cerebral symptoms were only mentioned in one instance, and they were those of hypochondriasis simply. In same cases observed by Dr. S. there was an absolute destruction or necrosis of liver-substance, indicating a diffuse inflammation, rather than one that had distinct walls and contained pus. Aspiration had been resorted to in two cases and incision in one.

Dr. Carpenter thought that statistics tended to disprove the position and relation between dysentery and abscess of the liver.

Dr. Satterthwaite replied that in this climate the rarity of the diseases precluded one from offering extended statistics. He believed that a casual relation existed often between hepatitis and dysentery, though in special epidemics the liver might not be implicated, as shown by Niemeyer during the last Franco-Prussian war.

The Society then went into Executive Session.—*Medical Record.*

